# Evidence of considerable C and N transfer from peas to cereals via direct root contact but not via mycorrhiza

**DOI:** 10.1038/s41598-021-90436-8

**Published:** 2021-06-01

**Authors:** Anke Hupe, Franziska Naether, Thorsten Haase, Christian Bruns, Jürgen Heß, Jens Dyckmans, Rainer Georg Joergensen, Florian Wichern

**Affiliations:** 1 Soil Biology and Plant Nutrition, University of Kassel, Nordbahnhofstr. 1a, 37213 Witzenhausen, Germany; 2 Organic Farming and Cropping, University of Kassel, Nordbahnhofstr. 1a, 37213 Witzenhausen, Germany; 3 Centre for Stable Isotope Research Analysis, University of Göttingen, Büsgenweg 2, 37077 Göttingen, Germany; 4 Soil Science and Plant Nutrition, Faculty of Life Sciences, Rhine-Waal University of Applied Sciences, Marie-Curie-Str. 1, 47533 Kleve, Germany; 5 Present Address: Landesbetrieb Landwirtschaft Hessen, Kassel, Germany

**Keywords:** Plant sciences, Stable isotope analysis

## Abstract

Intercropping of legumes and cereals is an important management method for improving yield stability, especially in organic farming systems. However, knowledge is restricted on the relevance of different nutrient transfer pathways. The objective of the study was to quantify nitrogen (N) and carbon (C) transfer from peas to triticale by (1) direct root contact (= R), (2) arbuscular mycorrhizal fungi (AMF; = A), and (3) diffusion (= D). Pea (*Pisum sativum* cv. Frisson and P2) and triticale (*Triticum* × *Secale* cv. Benetto) plants as intercrop were grown for 105 days. Treatment ADR enabled all transfer paths between the two crops. Treatment AD with root exclusion enabled AMF and diffusion transfer between peas and triticale. Treatment A with a diffusion gap barrier only allowed AMF transfer. Pea plants were labelled every 14 days with a ^13^C glucose and ^15^N urea solution, using the cotton wick technique. Direct root contact resulted in the highest pea rhizodeposition and thus the largest absolute amounts of N and C transfer to triticale. Root exclusion generally changed composition of rhizodeposits from fine root residues towards root exudates. Pea plant-N consisted of 17% N derived from rhizodeposition (NdfR) in treatment ADR but only 8% in the treatments AD and A, independently of pea variety, whereas pea plant-C consisted of 13% C derived from rhizodeposition (CdfR), without pea variety and transfer path treatment effects. Averaging all transfer path treatments, 6.7% of NdfR and 2.7% of CdfR was transferred from Frisson and P2 to triticale plants. Approximately 90% of this NdfR was transferred by direct root contact from Frisson to triticale and only 10% by AMF, whereas only 55% of CdfR was transferred to triticale by direct root contact, 40% by AMF and 5% by diffusion. Similar percentages were transferred from mutant P2 to triticale. Root exclusion generally changed RD composition from fine root residues towards root exudates.

## Introduction

N_2_ fixation by legumes is an important N input process in many farming systems, which can be improved by intercropping^1^. In this case, the cereal component is more competitive for soil inorganic N, forcing the legume crop to rely on nodulation and N_2_ fixation^2,3^. Pea (*Pisum sativum* L.) is a common legume partner for intercropping with cereals, particularly spring barley (*Hordeum vulgare* L.)^4,5^, spring wheat (*Triticum aestivum* L.)^6^, oats (*Avena sativa* L.)^1^, and triticale (*Triticum* × *Secale*)^6,7^. Intercropping of peas with cereals increases yield stability by reducing weed pressure and diseases^8^, but most likely also due to belowground nutrient transfer, e.g., by direct root contact, diffusion of exudates, and mycorrhiza^9^. Intercropping effects on rhizodeposition and soil microbial properties may also account for yield advantages^10^.

Plant rhizodeposits represent an essential C and N input into soil^11,12^. Consequently, the quantification of rhizodeposits improves N balance calculations^13^ and C sequestration estimates of crop rotations^11^. However, rhizodeposits not only consist of easily available C and N derived from root exudates, but also of relatively recalcitrant particulate components derived from decaying fine root remains^14^. Consequently, the term rhizodeposition should be used in its broad definition^11^, including all ions, gases, volatile organic components, exudates, lysates, and secretions released by living roots as well as decaying fine root fragments. The amount of rhizodeposits depends on species, root development, and growth stage. Flowering is usually followed by root decay, which increases the contribution of small particulate root fragments and root debris as well as sloughed cells to rhizodeposits^11,14^.

Another important control of rhizodeposits is the symbiosis with arbuscular mycorrhizal fungi (AMF)^14^. More than 80% of land plant species form AMF symbiosis, which can provide additional phosphorus, micronutrients and water to the host plants in exchange for assimilates. The resulting strong C flux into the mycorrhizosphere can amount to as much as 20% of assimilated C^15^. AMF also affects root architecture as well as quality and quantity of rhizodeposits^16,17^. Due to the low host specificity, AMF can form interspecies connections between intercropping partners as hosts^18,19^. However, knowledge on the amounts of C and N transferred by this AMF hyphal network is restricted in comparison with other transfer processes. The most important transfer paths are diffusion of soluble rhizodeposits through the soil solution^18^ and direct root contact^20,21^, i.e., the immediate uptake of rhizodeposits after release from neighbouring roots and AMF^18^.

Root exclusion with a nylon net barrier and solid diffusion barriers have recently been used for investigating AMF effects on N transfer between legume and non-legume crops^22,23^. However, a solid barrier additionally hinders the formation of AMF networks between these two types of crops, so that a gap barrier^18^, e.g., two nylon nets with a narrow air space, is more appropriate to separate a pure AMF effect from the combined AMF and diffusion effect on N transfer. In such an experiment, the AMF-resistant winter-pea (*Pisum sativum* L.) mutant P2^24^ can most likely be used as an effective additional zero-control in comparison with its isogenetic and symbiotic isoline Frisson. The often lower belowground biomass of mutant P2 in comparison with Frisson^17,25,26^ does not matter in this context, as the relative effects of root contact and diffusion are more important.

For quantifying the AMF contribution to N transfer processes between different plant species, mutant P2, Frisson and triticale as intercrop were grown in a pot experiment, using root exclusion nylon nets and gap diffusion barriers as additional treatments. The two pea varieties were both labelled with ^13^C and ^15^N using the cotton wick stem feeding technique^17,27^. The following two hypotheses were investigated: (1) Direct root contact with triticale results in the highest pea rhizodeposition, because the higher root competition between the plants increased root turnover. (2) AMF transfer of N and C from peas to triticale exceeds diffusion, but not that of direct root contact.

## Results

On average, 87% of the N tracer supplied to Frisson was recovered, which was more (*P* < 0.05, pairwise t-test) than the 67% found in mutant P2, however, without any effects of the transfer path treatments (Table 1). This difference is mainly caused by the lower ^15^N recovery in P2 straw, i.e., 25 versus 12% (*P* < 0.05, pairwise t-test), whereas most of the other fractions did not differ between the two pea varieties (*P* > 0.05, pairwise t-test). Approximately 16% was recovered in the labelling system, 35% in pea grain, 1.1% in pea roots and 4.4% in soil. In the Frisson treatment ADR, more ^15^N was recovered in the triticale roots in comparison with all other treatments, i.e., 0.27 versus 0.05% (*P* < 0.05; pairwise t-test).

**Table 1:** Total ^15^N recovery in the labelling systems and in pea plant parts supplied with the labelling solutions as well as in triticale plant parts as intercrop at the end of a 105-day greenhouse pot experiment; transfer paths: ADR = AMF, diffusion, and root contact between pea and intercrop; AD = AMF and diffusion; A = only AMF; CV = mean coefficient of variation between replicate pots (n = 6).

Plant	Transfer paths	^15^N recovery (%)						Triticale intercrop		
		Total	Labelling system	Pea						
				Grain	Straw	Roots	Soil	Soil	Roots	Straw
Frisson	ADR	89	15.8	36.8	22.9	0.7	6.0	6.0	0.27	0.6
	AD	87	23.1	32.8	24.5	2.0	4.3	0.2	0.11	0.2
	A	84	12.9	36.6	27.2	2.2	4.2	0.3	0.07	0.3
P2	ADR	70	15.9	33.5	11.4	0.4	4.0	4.0	0.06	0.4
	AD	67	11.5	37.5	13.4	0.6	3.3	0.4	0.02	0.3
	A	64	18.3	30.2	9.9	0.4	4.6	0.1	0.01	0.3
CV (± %)		14	45	28	25	54	41	94	91	58

On average, only 42% of the ^13^C tracer was recovered, without significant difference between the pea varieties (Table 2). Approximately 4.6% was recovered in the labelling system, 3.8% in the pea grain, 27.4% in the pea straw, and 2.6% in the soil. In the Frisson treatments AD and A, more ^13^C was recovered in the pea roots in comparison with all other treatments, i.e., on average 2.1 versus 0.8% (*P* < 0.05; pairwise t-test). In the Frisson treatment ADR, more ^13^C was recovered in the triticale roots in comparison with all other treatments, i.e., on average 0.19 versus 0.03% (*P* < 0.05, pairwise t-test).

**Table 2:** Total ^13^C recovery in the labelling systems and in pea plant parts supplied with the labelling solutions as well as in triticale plant parts as intercrop.

Plant	Transfer paths	^13^C recovery (%)						Triticale intercrop		
		Total	Labelling system	Pea						
				Grain	Straw	Roots	Soil	Soil	Roots	Straw
Frisson	ADR	38	4.7	4.7	22.3	0.9	2.3	2.3	0.19	0.094
	AD	42	5.8	3.6	27.1	1.6	2.7	1.2	0.06	0.001
	A	49	3.8	4.2	33.8	2.5	2.7	1.4	0.04	0.109
P2	ADR	37	5.0	0.3	26.4	0.8	2.2	2.2	0.05	0.033
	AD	45	4.1	5.7	30.5	1.0	2.3	1.7	0.01	0.058
	A	38	4.1	4.4	24.3	0.6	3.3	1.3	0.01	0.073
CV (± %)		18	43	28	24	29	41	93	72	38

On average, 16.6% of the Frisson roots were AMF colonized, 0.7% of the P2 and 9.7% of the triticale roots (results not shown). N plant^−1^ in Frisson AGP (aboveground plant biomass), roots, and rhizodeposits (RD) were on average three times higher than the respective fractions of P2 (Table 3). In the Frisson treatment ADR, root-N plant^−1^ reached only a third of the treatments AD and A, leading to a three times higher shoot-N/root-N ratio (*P* < 0.05, pairwise t-test). AGP-N, root-N and shoot-N/root-N ratio of triticale were not affected by pea varieties and transfer path treatments. Mean AGP-C of Frisson and P2 followed AGP-N, with C/N ratios of 13 and 20, respectively, as calculated from the data in Table 4. Mean root-C and RD-C of Frisson and P2 followed root-N and RD-N, with average C/N ratios of 17 and 24, respectively. Mean AGP-C and root-C of triticale followed AGP-N and root-N, resulting in C/N ratios of 9 and 22, respectively, without pea variety effects.

**Table 3:** Mean N amounts per plant in above-ground plant (AGP) biomass, root biomass, and rhizodeposition (RD) of two pea varieties and triticale as intercrop at the end of a 105-day greenhouse pot experiment; transfer paths: ADR = AMF, diffusion, and root contact between pea and intercrop; AD = AMF and diffusion; A = only AMF; CV = mean coefficient of variation between replicate pots (n = 6); different letters within a pea-specific treatment indicate a significant difference (protected LSD test; *P* < 0.05).

Plant	Transfer paths	Pea				Triticale intercrop			
		AGP-N	Root-N	RD-N	Shoot-N/	AGP-N		Root-N	Shoot-N/
		(mg plant^−1^)			root-N	(mg plant^−1^)			root-N
Frisson	ADR	294 a	2.9 b	68.3 a	182 a	145 a		8.4 a	21 a
	AD	411 a	8.1 a	33.2 ab	53 a	117 a		6.4 a	19 a
	A	324 a	7.8 a	24.0 b	51 a	125 a		6.5 a	20 a
P2	ADR	115 a	1.8 a	20.6 a	62 a	127 a		5.2 a	27 a
	AD	108 a	2.5 a	8.3 b	54 a	140 a		5.4 a	27 a
	A	81 a	2.0 a	9.8 b	46 a	129 a		7.1 a	20 a
CV (± %)		34	47	48	52	28		43	30

**Table 4:** Mean C amounts per plant in AGP, root biomass, and RD of two pea varieties and triticale as intercrop at the end of a 105-day greenhouse pot experiment.

Plant	Transfer paths	Pea				Triticale intercrop		
		AGP-C	Root-C	RD-C	Shoot-C/	AGP-C	Root-C	Shoot-C/
		(mg plant^−1^)			root-C	(mg plant^−1^)		root-C
Frisson	ADR	3870 b	49 b	784 a	134 a	1340 a	173 a	9 a
	AD	5550 a	134 a	830 a	44 b	1090 a	121 a	10 a
	A	4050 ab	126 a	443 a	37 b	1270 a	154 a	9 a
P2	ADR	2370 a	47 a	392 a	49 a	1170 a	109 a	12 a
	AD	2100 a	57 a	227 b	41 a	1300 a	126 a	11 a
	A	1700 a	42 a	265 ab	43 a	1220 a	161 a	9 a
CV (± %)		28	41	34	43	32	54	36

Microbial biomass nitrogen (MBN) and microbial biomass carbon (MBC) varied around mean contents of 29.3 and 197 µg g^−1^ soil, respectively, leading to an average MB-C/N ratio of 6.7, without pea variety and transfer path treatment effects on these three microbial indices, according to a two-way analysis of variance (Table 5). MBN contained 1.2% and MBC 2.0% dfR, averaging all three Frisson treatments, but only 0.3% and 0.8% dfR, respectively, averaging the P2 treatments. In the triticale treatments AD and A, the respective average percentages were only 0.07% and 0.12%. Inorganic N and extractable C varied around mean contents of 44 and 100 µg g^−1^ soil. Inorganic N contained 0.04% and extractable C 2.2% dfR, averaging all three Frisson treatments, but only 0.01% and 0.4% dfR, respectively, averaging the P2 treatments. In the triticale treatments AD and A, inorganic N and extractable C contained only traces of dfR.

**Table 5:** Mean microbial biomass N (MBN) and C (MBC) contents, mean MBN and MBC percentages derived from rhizodeposition (dfR) in soil cropped with two pea varieties and triticale as intercrop after a 105-days greenhouse pot experiment; transfer paths: ADR = AMF, diffusion, and root contact between pea and intercrop; AD = AMF and diffusion; A = only AMF; CV = mean coefficient of variation between replicate pots (n = 6); probability values of a two-way ANOVA, using pea variety and transfer path treatment as factors, NS = not significant.

Plants	Transfer paths	MBN		MBC		Inorganic N		Extractable C	
		(µg g^−1^ soil)	dfR (%)	(µg g^−1^ soil)	dfR (%)	(µg g^−1^ soil)	dfR (%)	(µg g^−1^ soil)	dfR (%)
**Frisson + triticale**	ADR	30.7	0.44	205	1.09	40	0.017	104	1.01
Frisson	AD	30.2	1.94	210	3.10	43	0.022	104	2.76
Frisson	A	28.4	1.15	196	1.80	53	0.050	98	1.61
Triticale	AD	31.3	0.14	210	0.08	38	0.002	107	0.09
Triticale	A	28.1	0.06	196	0.38	34	0.000	102	0.34
**P2 + triticale**	ADR	28.2	0.32	187	1.33	44	0.003	95	1.20
P2	AD	27.6	0.31	180	0.49	48	0.010	93	0.44
P2	A	27.9	0.33	186	0.50	58	0.006	93	0.45
Triticale	AD	30.8	0.05	204	0.02	49	0.001	102	0.02
Triticale	A	29.3	0.02	197	0.00	36	0.000	100	0.00
**Probability values**									
Plant		NS	< 0.01	< 0.01	< 0.01	NS	< 0.01	< 0.01	< 0.01
Transfer paths		NS	NS	NS	< 0.01	NS	NS	NS	< 0.01
Plant × transfer path		NS	< 0.01	NS	< 0.01	NS	< 0.01	NS	< 0.01
CV (± %)		9.6	80	7.0	96	32	92	6.8	89

Pea plant-N consisted of 17% NdfR in treatment ADR but only 8% in the treatments AD and A, independently of pea variety (Table 6). This difference was not reflected by the NdfR composition. Averaging all transfer path treatments with Frisson and P2 plants, 90% of this NdfR was found as soil N, 12% as MBN, and 0.42% as extractable inorganic N. In contrast, only 5% was recovered as soil N, 1.2% as MBN and virtually nothing as extractable inorganic N in the sole presence of triticale plants, averaging treatments AD and A. Pea plant-C consisted of 13% CdfR, without pea variety and transfer path treatment effects (Table 6). Even 96% of this CdfR was found as soil organic carbon (SOC) in treatment ADR with the combined presence of pea and triticale plants, 64% in the pea treatments AD and A, but only 34% in the triticale treatments AD and A. Averaging all transfer path treatments with Frisson and P2 plants, 6.7% of CdfR was found as MBC and 3.1% as extractable organic C. Only 0.5% of CdfR was recovered as MBC and 0.2% as extractable organic C in the sole presence of triticale plants, averaging treatments AD and A.

**Table 6:** Relative distribution of N and C derived from pea rhizodeposition in soil and triticale at the end of a 105-day greenhouse pot experiment; transfer paths: ADR = AMF, diffusion, and root contact between pea and intercrop; AD = AMF and diffusion; A = only AMF; CV = mean coefficient of variation between replicate pots (n = 6); probability values of a two-way ANOVA, using pea variety and transfer path treatment as factors, NS = not significant.

Varieties	Transfer	NdfR	% of NdfR in				CdfR	% of CdfR in			
	paths	(% pea-N)	Soil N^a^	MBN	Inorganic N	Triticale	(% pea-C)	SOC^a^	MBC	Extractable C	Triticale
**Frisson + triticale**	ADR	17.5	92.0	5.4	0.18	8.1	16.1	94.4	6.2	2.8	5.7
Frisson	AD	7.2	91.2	19.7	0.26		12.3	67.2	7.1	3.2	
Frisson	A	6.9	86.4	15.2	1.03		9.8	64.0	8.2	3.7	
Triticale	AD		3.1	1.1	0.02	5.7		31.4	0.2	0.1	1.4
Triticale	A		6.0	0.6	0.01	7.6		32.5	1.5	0.7	3.5
**P2 + triticale**	ADR	16.4	94.4	9.4	0.16	5.6	14.0	97.8	11.0	5.2	2.2
P2	AD	7.1	83.3	11.9	0.58		9.7	56.4	4.2	1.9	
P2	A	11.6	92.6	10.4	0.29		13.8	69.0	3.7	1.7	
Triticale	AD		9.5	2.1	0.07	7.2		42.1	0.2	0.1	1.5
Triticale	A		1.2	0.9	0.01	6.2		29.1	0.0	0.0	1.9
**Probability values**											
Plant		NS	< 0.01	< 0.01	< 0.01	NS	NS	< 0.01	< 0.01	< 0.01	NS
Transfer path		0.01	NS	< 0.01	0.02	NS	NS	NS	< 0.01	< 0.01	NS
Plant × transfer path		NS	< 0.01	< 0.01	< 0.01	NS	NS	< 0.01	< 0.01	< 0.01	NS
CV (± %)		39	44	69	79	53	29	14	76	78	140

^a^Including MBN and 0.5 K_2_SO_4_ extractable inorganic N or MBC and 0.5 K_2_SO_4_ extractable organic C from non-fumigated soils.

Averaging all transfer path treatments, 6.7% of NdfR and 2.7% of CdfR was transferred from Frisson and P2 to triticale plants. Approximately 90% of this NdfR was transferred by direct root contact from Frisson to triticale and only 10% by AMF, whereas only 55% of CdfR was transferred to triticale by direct root contact, 40% by AMF and 5% by diffusion. Similar percentages were transferred from mutant P2 to triticale.

## Discussion

### Direct root contact effects on rhizodeposition

Direct root contact between the two pea varieties and triticale in treatment ADR strongly increased the amounts of pea RD-N but also to some extent RD-C in comparison with the transfer path treatments AD and A. In contrast to the total amounts, direct root contact had no effect on the pea RD percentages taken up by triticale plants. On average, 6.7% pea RD-N and 2.7% pea RD-C were found in triticale, regardless of pea variety and transfer path treatment. In agreement with the current data, N transfer rates of between 2.4 and 4.7% have been observed from alfalfa (*Medicago sativa* L.) to maize (*Zea mays* L.), grown in large pots (51 kg soil in 67.5 dm^−3^) for 80 days under ambient conditions^23^. Similarly, N transfer rates of between 3.1 and 6.0% have been estimated from soybean (*Glycine max* L.) to maize^22^, grown in pots (1.4 kg soil) for 58 days. A mean N transfer rate of 8.4%, with a range of 2.7 to 22.5%, has been reported from N_2_-fixing legumes to non-N_2_-fixing mycorrhizal cereals^18^, excluding extreme values obtained from plants not commonly used as crops. A mean N transfer rate of 16% was measured from faba bean (*Vicia faba* L.) to intercropped durum wheat (*Triticum durum* Desf.)^28^, using the ^15^N isotope dilution method. In contrast, transfer rates between 2.0 and 2.7% were observed from faba bean to durum wheat^29^, using a stem feeding technique.

The current results indicate that the amount of N and C transferred from pea to triticale increased with the absolute amount of pea rhizodeposition, due to the larger root surface and contact areas between the intercropping partners in treatment ADR. Root development of strongly growing mycorrhizal Frisson suffered from the competition with mycorrhizal triticale roots, whereas root development of weakly growing non-mycorrhizal mutant P2 roots were promoted by the presence of triticale roots. In contrast, it has also been observed that mycorrhization favoured durum wheat^29^, without negatively affecting faba bean as intercrop. This suggests that growth-related interactions between legumes and cereals differ between varieties.

An important reason for the strong effects of direct root contact are resource complementarity between cereals and legumes. These two intercropping partners use different chemical nutrient forms, which facilitates the interspecific nutrient uptake indirectly by RD-induced microbial activity and directly by specific root exudates^30,31^. An example of this direct interspecific facilitation of nutrient uptake is the effect of phytosiderophores such as deoxymugineic acid secreted by monocotyledonous cereals into the rhizosphere^32^. They desorb for example otherwise insoluble Fe and Zn forms in soil, which can also be taken up by dicotyledonous legumes unable to secrete the respective phytosiderophores^32^. Triticale might also be able to excrete phytohormones such as strigolactones^33^, which affect root branching and AMF colonization only over short distances. However, the role of such secondary plant components released by the intercrops to stimulate the release of legume rhizodeposits needs more experimental evidence.

### Triticale effects on microbial pea root turnover

In treatment ADR, triticale roots passively consumed available RD-N and RD-C and induced an increased turnover of Frisson roots. This increase led to lower presence of pea root-C at harvest, accompanied by RD-C losses as CO_2_, as indicated by the roughly halved RD-C/N ratio in comparison with all P2 treatments and Frisson treatments AD and A. Due to these different treatment effects on RD-N and RD-C, a higher relative contribution of mycorrhizal transfer from Frisson to triticale was calculated for RD-C than for RD-N. Different responses of RD-N and RD-C to growth conditions have already been emphasized, comparing pot and field experiments^14^. An attractive suggestion is the possibility of using the RD-C/root C ratio obtained in a pot experiment to estimate RD-C under field conditions by solely assessing root DW^12^. However, this approach should be used cautiously, as the RD-C/root-C and RD-N/root-N ratios as well as the RD-C/N ratio are not necessarily constant during all growth conditions and may vary, especially comparing pot and field conditions^14^.

In treatment ADR with direct root contact, the RD-N/root-N and RD-C/root-C ratios were 18 and 12, respectively, averaging the results of both pea varieties. The corresponding ratios were markedly lower, i.e., 4 and 5, respectively, in treatments AD and A. The RD-N/root-N and RD-C/root-C ratios suggest a higher contribution of fine root residues to pea RD in comparison with treatments AD and A. More root residues must be combined with a higher fibre concentration^34,35^. This would explain why the presence of triticale roots also decreased the incorporation of RD-N and RD-C into the soil microbial biomass, due to increased recalcitrance. This shift in RD composition should be reflected by a higher contribution of root-derived particulate organic matter^4^ and a higher contribution of saprotrophic fungi, specialized in the breakdown of plant fibres^36^. Other possibilities are that the uptake of pea RD by triticale roots might reduce the availability to soil microorganisms or that triticale RD might dilute the effects of pea RD. The exact reasons need more specific attention in future studies.

### Remarks on labelling, fertilization, and inoculation

The cotton wick technique allows continuous solution uptake from a vial, delivering the tracer solution continuously to all plant organs, i.e., also to the roots^37^. The general recovery of ^15^N and ^13^C is lower than that of an experiment^17^, using similar experimental conditions. However, the recovery in soil and roots was roughly the same^17^, giving confidence that the current RD-N and RD-C data were not affected by any methodological bias. In contrast, the current recovery in plant tissue and in labelling system are both roughly 15% lower. Gaseous losses and sample inhomogeneity might have contributed to the lower recovery rates. A finally 20% higher recovery of ^15^N in Frisson than in P2 plant material was also previously observed for unknown reasons^17^.

Pea variety or transfer path treatments did not affect N-transfer from peas to triticale and also not triticale yield indices. One reason might be the good nutrient supply by the soil. Another reason might be insufficient vernalisation, which kept the triticale plants in the vegetative state. Consequently, the N-transfer from peas to triticale might be underestimated in this pot trial. To ensure similar growth conditions for the non-nodulating and non-mycorrhizal mutant P2, NPK fertilizer was applied at the beginning of the pot experiment. However, these conditions most likely had negative effects on mycorrhizal C and N transfer^2,23^. Future studies should elucidate nutrient status effects on the transfer of N between legumes and cereals.

Although inoculated with AMF spores, the mycorrhizal root colonisation of Frisson plants and triticale was relatively low in the current pot experiment compared with field-grown peas and barley, which was approximately 60% at flowering and 30% at late booting, respectively^4^. However, the mycorrhizal root colonisation between 17 and 10% is in line with several pot experiments that used pea species as experimental plants^17,25,26^. A 43% mycorrhizal root colonisation of Frisson pea plants was measured at flowering^26^, which declined to 10% at harvest. Similarly, maximum values of 32% for mycorrhizal root colonisation of Frisson pea plants was observed at flowering and 17% minimum values at dry ripeness^17^. The changes in mycorrhizal root colonization during maturation^4^ are accompanied by strong shifts in the root colonizing fungal microbiome^4,38^, e.g., an increasing presence of saprotrophic but also pathogenic fungi^39^. The presence of triticale roots might have affected the pea root turnover in an unknown way.

### Distribution of rhizodeposition in soil fractions

A striking feature is the similar relative distribution of labelled NdfR and CdfR in the different soil fractions, i.e., soil N, MBN, and inorganic N, SOC, MBC, and extractable C, with minor differences between Frisson and mutant P2. The difference in plant development between the two isolines, usually emphasized as a problem^17,26^, did not affect the relative distribution of labelled NdfR and CdfR in soil between the two pea varieties.

The transfer path treatments showed only minor effects on the relative distribution of labelled NdfR in all fractions, whereas CdfR differed strongly between treatment ADR and treatments AD and A. Under the two pea varieties, roughly 30% less labelled CdfR was found as SOC than NdfR as soil N in treatments AD and A in comparison with treatment ADR. The reverse was true under triticale. Consequently, diffusion apparently played a negligible role for NdfR and a slightly larger role for CdfR. However, in contrast to nitrate, soluble organic C components cannot be transferred over large distances by diffusion^40^. This suggests that triticale AMF collected organic ^15^N and especially ^13^C containing pea RD-N and RD-C components by diffusion, without being directly connected to the pea plants. This is clearly demonstrated by the similar relative distribution of labelled NdfR and CdfR fractions between mycorrhizal Frisson and its non-mycorrhizal mutant P2. This uptake process, called “cheating”^41^, allows AMF to survive without a host in soil^42^.

## Conclusions

All treatments had only minor effects on pea and triticale aboveground and root biomass, most likely due to N fertilization of all treatments. Direct root contact of the winter pea varieties Frisson and its non-nodulating isogenetic mutant P2 with triticale resulted in the highest pea rhizodeposition. This increase was accompanied by a decrease in root biomass for Frisson but not for mutant P2, indicating an increased root turnover of the wild type by competing with triticale roots. Root exclusion generally changed RD composition from fine root residues towards root exudates. In this case, AMF transferred significant amounts of N and C from the peas to triticale, but this transfer path was much smaller than that of direct root contact, especially for N. A significant N transfer of pea rhizodeposition to triticale by diffusion was not detected, whereas small amounts of C were transferred by this process.

## Methods

### Soil

The arable soil used in the greenhouse experiment was collected at 0–30 cm depth from the research station of the University of Kassel, which is located near Neu-Eichenberg, Northern Hesse, Germany (N 51°22′48.139", E 9°54′42.120", 220 m asl). The field had been cultivated according to organic farming practices for 25 years. The preceding crop was barley (*Hordeum vulgare* L.) variety Marthe. The soil was sieved (< 10 mm) and stored for 6 weeks before the experiment started in boxes at 10 to 15 °C in the dark. The silty loam was classified as a Haplic Luvisol^43^. Soil texture was 13% clay, 83% silt, and 3% sand. Soil pH-CaCl_2_ was 6.0; SOC and total N contents were 12 mg and 1.3 mg g^−1^ soil, respectively.

### Experimental design

The 105-day pot experiment was carried out in a greenhouse at the University of Kassel, Faculty of Organic Agricultural Sciences in Witzenhausen, Northern Hesse, Germany. Our study protocol complies with relevant institutional, national, and international guidelines and legislation.

In each pot, a semi-leafless winter pea (*Pisum sativum* L.) plant, variety Frisson or its non-mycorrhizal (myc^−^) and non-nodulating (nod^−^) isogenetic mutant P2^24^, were grown with two plants of triticale (*Triticum* × *Secale* Wittm. ex. A. Camus) variety Benetto as intercrop. We obtained permission to collect winter pea plant (Pisum sativum) material from the seed manufacturers and providers. The relative humidity was set at 60% and light intensity at 110 klxh d^−1^. Temperature was adjusted to 12 h at 20 °C during the day and at 15 °C during the night from August to October. The respective temperatures were 15 °C and 10 °C from October until December.

The pots of 8.5 l volume (28 cm diameter and 15 cm height) were filled with 11.05 kg soil and compacted to a bulk density of 1.3 g cm^−3^. One day before seeding, Ca was applied at a rate of 100 kg ha^−1^. On the day of seeding, fertilizers were added equivalent to 150 kg N, 17 kg P, 43 kg K, and 9 kg Mg ha^−1^. This was done to suppress nodulation of Frisson^25^ and to reduce differences in plant dry weight (DW) between Frisson and P2^44^. All pots adjusted to a water holding capacity between 60 and 80% by weekly adding demineralized water after weighing. The pots were placed in a randomized block design with 6 replicates for each treatment and each pea variety. Pot positioning was newly randomized every week to minimize the possible influence of greenhouse microclimate. All pots were AMF inoculated with 5 g rootgrow professional (PlantWorks, Sittingbourne, UK), placed directly below the seeds to ensure symbiosis with Frisson.

**Figure 1:**
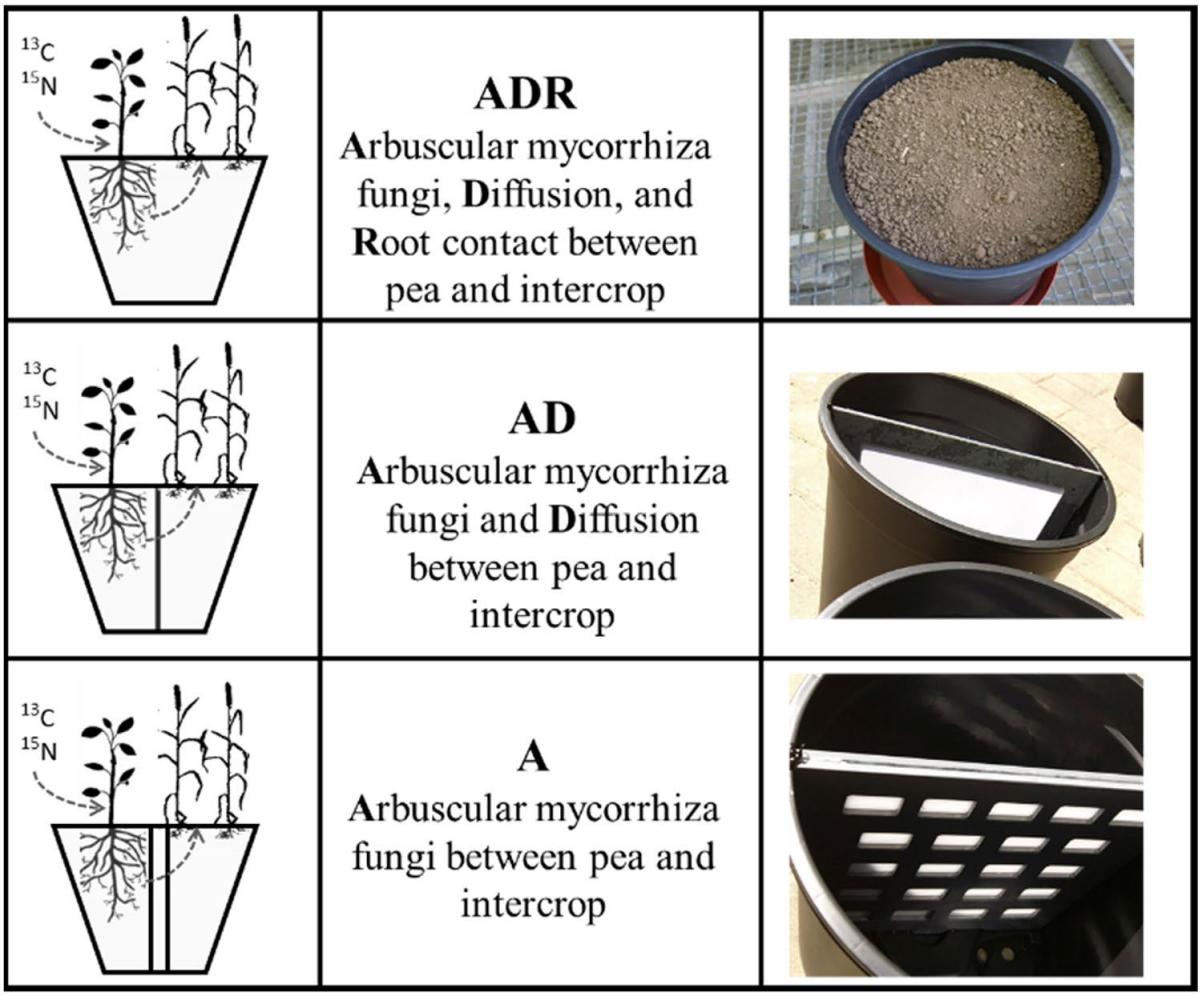
Schematic setting of pot trial with three treatments and the symbols used for the treatments; transfer paths: ADR = AMF, diffusion, and root contact between pea and intercrop; AD = AMF and diffusion; A = only AMF.

In the first treatment ADR, normal pots were used to enable all possible transfer paths, i.e., by AMF, diffusion, and direct root contact (Fig. 1). In the second treatment AD, a mesh of 30 µm stabilized by a polyethylene frame was installed in the pots to exclude direct root contact, reducing the transfer paths to AMF and diffusion. In the third treatment A, two parallel meshes on two polyethylene frames with a gap of 1 mm served as a diffusion barrier, allowing only AMF hyphae to pass the gap. This treatment served in combination with non-mycorrhizal P2 as a double-negative control.

### Labelling procedure

To trace C and N, pea plants were ^13^C and ^15^N labelled using a stem-feeding technique^45^ with solutions of 0.5% ^15^N-urea (95 Atom%) and 2% ^13^C-glucose (99 atom%) as previously described in detail^17^. Labelling began at the three-leaf stage and a slow but steady uptake of solution was observed. The isotope solution was refilled every 2 weeks, i.e. four times, which provides an approximation to continuous labelling^11,17^, essential for a homogeneous labelling of the plant roots.

Due to callus formation, the hole for the cotton wick was re-drilled before every labelling event. The time required for solution uptake varied. Pea plants needed at least 24 to 48 h to take up 1 ml solution. After solution uptake, the empty vials were filled with 0.5 ml deionized water after every labelling event to encourage complete solution uptake. However, solution uptake was often incomplete, so that the residual solution remained in the vials until the next labelling event. For estimating isotope recovery, vials and labelling systems were extracted with 200 ml 0.05 M K_2_SO_4_, followed by determination of organic C and total N in the extracts as described below. The recovered labels were calculated, assuming that the C and N were solely derived from the labelling solution.

Pots were covered with mesh (1 mm) to prevent soil contamination from falling leaves. For measuring the natural abundance of ^13^C and ^15^N in the pea plants, as background values for calculating rhizodeposition, 6 unlabelled control plants of Frisson and P2 were cultivated and randomized with the labelled pea plants^17^. It has been shown that the labelling system does not influence plant weight or plant development^46^. Therefore, plants for background values of the isotope ratio of ^13^C/^12^C and ^15^N/^14^N do not need a labelling system. Pea plants also had two growth support canes in their vessels. Because of an infestation with powdery mildew at the end of September and beginning of October, three applications of elemental sulphur were sprayed onto the plants.

### Plant and soil sampling

At harvest, vials and wicks were carefully removed and plants were cut off directly above the soil surface, immediately separated into stem, leaves, and grain, followed by drying at 60 °C for at least 48 h to determine DW. The soil was sieved (< 2 mm) and all visible roots were manually collected, washed with distilled water and dried at 60 °C, except an aliquot of 0.5 g fresh root material for the determination of mycorrhizal colonization. Soil was dried at 105 °C for approximately 24 h. Sub-samples of dried plant material and root-free soil material were homogenised in a ball mill. Total C and N concentrations as well as ^13^C/^12^C and ^15^N/^14^N ratios were analysed on an isotope ratio mass spectrometer (Finnigan MAT, Bremen, Germany) coupled to an elemental analyser (EA-IRMS).

### Microbial indicators

Mycorrhizal colonization of roots was determined by the gridline intersection method^47^ in 1.5 g fresh root material after staining with 0.1% trypan blue in 90% lactic acid^25^.

MBC and MBN were estimated by fumigation extraction^48,49^, including pre-extraction to remove living roots^50^. For pre-extraction, 30 g of field moist soil was horizontally shaken for 30 min with 70 ml 0.05 M K_2_SO_4_ at 200 rev min^−1^. Then, the soil suspension was centrifuged for 10 min at 4000 g and 6 °C. The supernatant was filtered and frozen for measuring organic C, total N, inorganic N, and the isotope ratios in the extracts. After pre-extraction, 15 g of the 0.05 M K_2_SO_4_ saturated soil was fumigated for 24 h at 25 °C with ethanol-free CHCl_3_. After CHCl_3_ removal, the fumigated and 15 g of the non-fumigated pre-extracted soil were further extracted with 60 ml 0.05 M K_2_SO_4_ and the extract kept frozen until further analyses. Organic C and total N in all extracts were analysed using a CN Analyser (Multi N/C 2100S, Analytik Jena, Germany). After freeze-drying of extracts, the ^13^C/^12^C and ^15^N/^14^N isotope ratios were determined by EA-IRMS.

### Calculation and statistical analyses

MBC was calculated as *E*_C_/*k*_EC_, where *E*_C_ = (organic C extracted from fumigated soils) − (organic C extracted from non-fumigated soils) and *k*_EC_ = 0.45^51^. MBN was calculated as *E*_N_/*k*_EN_, where *E*_N_ = (total N extracted from fumigated soils) − (total N extracted from non-fumigated soils) and *k*_EN_ = 0.54^48^.

The ^13^C_tracer_ derived from rhizodeposition (= ^13^C_tracer_dfR) and ^15^N_tracer_dfR were calculated using the mass balance approach (Eqs. 1 and 2)^52^:

1 $$^{{{13}}} {\rm C}_{{\text{tracer }}} \;{\rm dfR }\left( \% \right) = \frac{{^{{{13}}} {\rm C}_{{\text{tracer }}} \;{\rm dfR}}}{{^{{{13}}} {\rm C}_{{\text{tracer }}} \;{\rm in}\;{\text{total}}}} \times 100 $$

2 $$^{{{15}}} {\rm N}_{{\text{tracer }}} \;{\text{dfR }}\left( \% \right) = \frac{{^{{{15}}} {\rm N}_{{\text{tracer }}} \;{\text{dfR}}}}{{^{{{15}}} {\rm N}_{{\text{tracer }}} \;{\rm in}\;{\text{total}}}} \times 100 $$

It was assumed that the distribution of ^13^C_tracer_ and ^15^N_tracer_ in the plant corresponds to the distribution of total plant C and N uptake, consequently ^13^C_tracer_dfR (%) = CdfR (%) and ^15^N_tracer_dfR (%) = NdfR (%). CdfR and NdfR in the soil were separately calculated for MBC, MBN, SOC, total N and inorganic N^52^.

The N and C transfer pathways from Frisson to intercrop triticale (Tri) were calculated as % of N or C rhizodeposition (RD) recovered in triticale plant and soil (Eqs. 3, 4 and 5), where RD_Tri_ of treatment ADR was the amount of C uptake from pea RD:

3 $$ {\text{Root}}\;{\text{contact}}\;{\text{C }} = { }\frac{{\left( {RD_{Tri} \;of\;Treat\;ADR \left[ {{\text{mg}}\;pea\;C^{ - 1} } \right]} \right) - \left( {RD_{Tri} \;of\;Treat\;AD \left[ {{\text{mg}} \;pea\;C^{ - 1} } \right]} \right)}}{{\left( {RD_{Tri} \;of\;Treat\;ADR \left[ {{\text{mg}}\;pea\;C^{ - 1} } \right]} \right)}} \times 100 $$

4 $$ {\text{Diffusion}}\;{\text{C}} = { }\frac{{\left( {RD_{Tri} \;of\;Treat\;AD \left[ {{\text{mg}}\;pea\;C^{ - 1} } \right]} \right) - \left( {RD_{Tri} \;of\;Treat\;A \left[ {{\text{mg}}\;pea\;C^{ - 1} } \right]} \right) }}{{\left( {RD_{Tri} \;of\; Treat\;ADR \left[ {{\text{mg}}\;pea\;C^{ - 1} } \right]} \right)}} \times 100 $$

5 $$ {\text{AMF}}\;{\text{C}} = { }\frac{{\left( {RD_{Tri} \;of\;Treat\;A \left[ {{\text{mg}}\;pea\;C^{ - 1} } \right]} \right) }}{{\left( {RD_{Tri} \;of\;Treat\;ADR \left[ {{\text{mg}}\;pea\;C^{ - 1} } \right]} \right)}} \times 100 $$

Data presented in Tables and Figures are arithmetic means on a DW basis. Data were tested for normality of residue distribution and homogeneity of variances, using the Shapiro–Wilk and Levene test, respectively. If these prerequisites were violated, data were log transformed prior to analysis of variance (ANOVA). Plant parameters in Tables 3 and 4 were analysed by a pea variety-specific one-way ANOVA with the separation treatments as factors, followed by the protected LSD post-hoc test (*P* < 0.05). Rhizodeposition parameters in Tables 5 and 6 were analysed by a two-way ANOVA with the separation treatments and pea varieties as factors. In some cases, the Student t-test was performed on pairs of means to determine differences between Frisson and mutant P2 or treatments. All statistical analysis was performed using SPSS 22 (IBM, Armonk, US).

## Supplementary Information

Supplementary Information 1.

## References

[1] Neugschwandter RW, Kaul HP (2014). Sowing ratio and N fertilization affect yield and yield components of oat and pea in intercrops. Field Crops Res..

[2] Hu F, Tan Y, Yu A, Zhao C, Coulter JA, Fan Z, Yin W, Fan H, Chai Q (2018). Low N fertilizer application and intercropping increases N concentration in pea (*Pisum sativum* L.) grains. Front Plant Sci..

[3] Jensen ES, Carlsson G, Hauggaard-Nielsen H (2020). Intercropping of grain legumes and cereals improves the use of soil N resources and reduces the requirement for synthetic fertilizer N: a global-scale analysis. Agron. Sustain. Dev..

[4] Jannoura R, Joergensen RG, Bruns C (2014). Organic fertilizer effects on growth, crop yield, and soil microbial biomass indices in sole and intercropped peas and oats under organic farming conditions. Eur. J. Agron..

[5] Darch T, Giles CD, Blackwell MSA, George TS, Brown LK, Menezes-Blackburn D, Shand CA, Stutter MI, Lumsdon DG, Mezeli MM, Wendler R, Zhang H, Wearing C, Cooper P, Haygarth PM (2018). Inter- and intra-species intercropping of barley cultivars and legume species, as affected by soil phosphorus availability. Plant Soil.

[6] Monti M, Pellicanò A, Santonoceto C, Preiti G, Pristeri A (2016). Yield components and nitrogen use in cereal-pea intercrops in Mediterranean environment. Field Crops Res..

[7] Scalise A, Pappa VA, Gelsomino A, Rees RM (2017). Pea cultivar and wheat residues affect carbon/nitrogen dynamics in pea-triticale intercropping: a microcosms approach. Sci. Tot. Environ..

[8] Bedoussac L, Journet EP, Hauggaard-Nielsen H, Naudin C, Corre-Hellou G, Jensen ES, Prieur L, Justes E (2015). Ecological principles underlying the increase of productivity achieved by cereal-grain legume intercrops in organic farming. A review. Agron. Sustain. Dev..

[9] Garcia K, Doidy J, Zimmermann SD, Wipf D, Courty PE (2016). Take a trip through the plant and fungal transportome of mycorrhiza. Trends Plant Sci..

[10] Oelbermann M, Regehr A, Echarte L (2015). Changes in soil characteristics after six seasons of cereal–legume intercropping in the Southern Pampa. Geoderma Reg..

[11] Wichern F, Eberhardt E, Mayer J, Joergensen RG, Müller T (2008). Nitrogen rhizodeposition in agricultural crops: methods, estimates and future prospects. Soil Biol. Biochem..

[12] Pausch J, Tian J, Riederer M, Kuzyakov Y (2013). Estimation of rhizodeposition at field scale: upscaling of a ^14^C labeling study. Plant Soil.

[13] Fustec J, Lesuffleur F, Mahieu S, Cliquet JB (2010). Nitrogen rhizodeposition of legumes. A review. Agron. Sustain. Dev..

[14] Hupe A, Schulz H, Bruns C, Haase T, Heß J, Dyckmans J, Joergensen RG, Wichern F (2019). Get on your boots: estimating root biomass and rhizodeposition of peas under field conditions reveals the necessity of field experiments. Plant Soil.

[15] Parniske M (2008). Arbuscular mycorrhiza: the mother of plant root endosymbioses. Nat. Rev. Microbiol..

[16] Jones DL, Hodge A, Kuzyakov Y (2004). Plant and mycorrhizal regulation of rhizodeposition. New Phytol..

[17] Hupe A, Schulz H, Näther F, Bruns C, Haase T, Heß J, Joergensen RG, Wichern F (2018). Even flow? Changes of carbon and nitrogen release from pea roots over time. Plant Soil.

[18] He X, Xu M, Qiu CY, Zhou J (2009). Use of ^15^N stable isotope to quantify nitrogen transfer between mycorrhizal plants. J. Plant Ecol..

[19] Pepe A, Giovannetti M, Sbrana C (2018). Lifespan and functionality of mycorrhizal fungal mycelium are uncoupled from host plant lifespan. Sci. Rep..

[20] Xiao Y, Li L, Zhang F (2004). Effect of root contact on interspecific competition and N transfer between wheat and faba bean using direct and indirect ^15^N techniques. Plant Soil.

[21] Thilakarathna MS, McElroy MS, Chapagain T, Papadopoulos YA, Raizada MN (2016). Belowground nitrogen transfer from legumes to non-legumes under managed herbaceous cropping systems. A review. Agron. Sustain. Dev..

[22] Meng L, Zhang A, Wang F, Han X, Wang D, Li S (2015). Arbuscular mycorrhizal fungi and rhizobium facilitate nitrogen uptake and transfer in soybean/maize intercropping system. Front Plant Sci..

[23] Shao Z, Wang X, Gao Q, Zhang H, Yu H, Wang Y, Zhang J, Nasar J, Gao Y (2020). Root contact between maize and alfalfa facilitates nitrogen transfer and uptake using techniques of foliar ^15^N-labeling. Agronomy.

[24] Duc G, Trouvelot A, Gianinazzi-Pearson V, Gianinazzi S (1989). First report of non-mycorrhizal plant mutants (Myc−) obtained in pea (*Pisum sativum* L.) and fababean (*Vicia faba* L.). Plant Sci..

[25] Kleikamp B, Joergensen RG (2006). Evaluation of arbuscular mycorrhiza with symbiotic and nonsymbiotic pea isolines at three sites in the Alentejo, Portugal. J. Plant Nutr. Soil Sci..

[26] Jannoura R, Kleikamp B, Dyckmans J, Joergensen RG (2012). Impact of pea growth and of arbuscular mycorrhizal fungi on the decomposition of ^15^N-labeled maize residues. Biol. Fertil. Soils.

[27] Chalk PM, Peoples MB, McNeill AM, Boddey RM, Unkovich MJ, Gardener MJ, Silva CF, Chen D (2014). Methodologies for estimating nitrogen transfer between legumes and companion species in agro-ecosystems: a review of ^15^N-enriched techniques. Soil Biol. Biochem..

[28] Wahbi S, Maghraoui T, Hafidi M, Sanguin H, Oufdou K, Prin Y, Duponnois R, Galiana A (2016). Enhanced transfer of biologically fixed N from faba bean to intercropped wheat through mycorrhizal symbiosis. Appl. Soil Ecol..

[29] Ingraffia R, Amato G, Frenda AS, Giambalvo D (2019). Impacts of arbuscular mycorrhizal fungi on nutrient uptake, N_2_ fixation, N transfer, and growth in a wheat/faba bean intercropping system. PLoS ONE.

[30] Fusconi A (2014). Regulation of root morphogenesis in arbuscular mycorrhizae, what role do fungal exudates, phosphate, sugars and hormones play in lateral root formation. Ann. Bot..

[31] Wang W, Shi J, Xie Q, Jiang Y, Yu N, Wang E (2017). Nutrient exchange and regulation in arbuscular mycorrhizal symbiosis. Mol. Plant.

[32] Xue Y, Xia H, Christie P, Zhang Z, Li L, Tang C (2016). Crop acquisition of phosphorus, iron and zinc from soil in cereal/legume intercropping systems: a critical review. Ann. Bot..

[33] Abdelhalim T, Jannoura R, Joergensen RG (2019). Mycorrhiza response and phosphorus acquisition efficiency of sorghum cultivars differing in strigolactone composition. Plant Soil.

[34] Louarn G, Pereira-Lopès E, Fustec J, Mary B, Voisin AS, de Carvalho PCF, Gastal F (2015). The amounts and dynamics of nitrogen transfer to grasses differ in alfalfa and white clover-based grass-legume mixtures as a result of rooting strategies and rhizodeposit quality. Plant Soil.

[35] Faust S, Kaiser K, Wiedner K, Glaser B, Joergensen RG (2018). Comparison of different methods to determine lignin concentration and quality in herbaceous and woody plant residues. Plant Soil.

[36] Baldrian P, Voříšková J, Dobiášová P, Merhautová V, Lisá L, Valášková V (2011). Production of extracellular enzymes and degradation of biopolymers by saprotrophic microfungi from the upper layers of forest soil. Plant Soil.

[37] Wichern F, Andreeva D, Joergensen RG, Kuzyakov Y (2011). Distribution of applied ^14^C and ^15^N in legumes using two different labelling methods. J. Plant Nutr. Soil Sci..

[38] Turner TR, Ramakrishnan K, Walshaw J, Heavens D, Alston M, Swarbreck D, Osbourn A, Grant A, Poole PS (2013). Comparative metatranscriptomics reveals kingdom level changes in the rhizosphere microbiome of plants. ISME J..

[39] Yu L, Nicolaisen M, Larsen J, Ravnskov S (2012). Molecular characterization of root-associated fungal communities in relation to health status of *Pisum sativum* using barcoded pyrosequencing. Plant Soil.

[40] Gunina A, Kuzyakov Y (2015). Sugars in soil and sweets for microorganisms: review of origin, content, composition and fate. Soil Biol. Biochem..

[41] Allison SD (2005). Cheaters, diffusion and nutrients constrain decomposition by microbial enzymes in spatially structured environments. Ecol. Lett..

[42] Joergensen RG, Wichern F (2018). Alive and kicking: why dormant soil microorganisms matter. Soil Biol. Biochem..

[43] IUSS Working Group. WRB World reference base for soil resources 2014 (update 2015), international soil classification system for naming soils and creating legends for soil maps. World Soil Resources Reports (2015).

[44] Mahieu S, Fustec J, Jensen ES, Crozat Y (2009). Does labelling frequency affect N rhizodeposition assessment using the cotton-wick method?. Soil Biol. Biochem..

[45] Russell CA, Fillery IRP (1996). Estimates of lupin below-ground biomass nitrogen, drymatter, and nitrogen turnover to wheat. Crop Pasture Sci..

[46] Wichern F, Mayer J, Joergensen R, Müller T (2010). Evaluation of the wick method for in situ ^13^C and ^15^N labelling of annual plants using sugar-urea mixtures. Plant Soil.

[47] Phillips JM, Hayman DS (1970). Improved procedures for clearing roots and staining parasitic and vesicular-arbuscular mycorrhizal fungi for rapid assessment of infection. Transact. Brit. Mycol. Soc..

[48] Brookes PC, Landman A, Pruden G, Jenkinson DS (1985). Chloroform fumigation and the release of soil nitrogen. A rapid direct extraction method to measure microbial biomass nitrogen in soil. Soil Biol. Biochem..

[49] Vance ED, Brookes PC, Jenkinson DS (1987). An extraction method for measuring soil microbial biomass C. Soil Biol. Biochem..

[50] Mueller T, Joergensen RG, Meyer B (1992). Estimation of soil microbial biomass C in the p resence of living roots by fumigation-extraction. Soil Biol. Biochem..

[51] Wu J, Joergensen RG, Pommerening B, Chaussod R, Brookes PC (1990). Measurement of soil microbial biomass C by fumigation-extraction—an automated procedure. Soil Biol. Biochem..

[52] Hupe A, Schulz H, Bruns C, Joergensen RG, Wichern F (2016). Digging in the dirt—inadequacy of below-ground plant biomass quantification. Soil Biol. Biochem..

