# Supplementary material for: Evidence of considerable C and N transfer from peas to cereals via direct root contact but not via mycorrhiza
Source: Sci Rep. 2021 Jun 1;11:11424. doi: 10.1038/s41598-021-90436-8 (PMC8169652; doi:10.1038/s41598-021-90436-8)
Supplement: Supplementary file 1 — Supplementary Information 1. [file 41598_2021_90436_MOESM1_ESM.docx]

Evidence of considerable C and N transfer from peas to cereals via direct root contact but not via mycorrhiza

**Anke Hupe^1,2^^[[1]](#footnote-1)^*, Franziska Naether^2^, Thorsten Haase^2^**, Christian Bruns^2^, Jürgen Heß^2^, Jens Dyckmans^3^, Rainer Georg Joergensen^2^ & Florian Wichern^4^**

^1^ Soil Biology and Plant Nutrition, University of Kassel, Nordbahnhofstr. 1a, 37213 Witzenhausen Germany

^2^ Organic Farming and Cropping, University of Kassel, Nordbahnhofstr. 1a, 37213 Witzenhausen, Germany

^3^ Centre for Stable Isotope Research Analysis, University of Göttingen,
Büsgenweg 2, 37077 Göttingen, Germany

^4^ Soil Science and Plant Nutrition, Faculty of Life Sciences, Rhine-Waal University of Applied Sciences, Marie-Curie-Str. 1, 47533 Kleve, Germany

Supplementary Table S1a. Enrichment with ^15^N in pea plant parts supplied with the labelling solutions, and in triticale plant parts as intercrop at the end of a 105-day greenhouse pot experiment; transfer paths: ADR = AMF, diffusion, and root contact between pea and intercrop; AD = AMF and diffusion; A = only AMF.; CV = mean coefficient of variation between replicate pots (n = 6).

|  | Transfer | ^15^N atom%excess | | |  |  |  | | |
| --- | --- | --- | --- | --- | --- | --- | --- | --- | --- |
| Varieties | path | Pea | | |  |  | Triticale intercrop | | |
|  |  | Grain | Straw | Roots | Soil |  | Soil | Roots | Straw |
| Frisson | ADR | 1.4808 | 3.2080 | 1.9863 | 0.0034 |  | 0.0034 | 0.1183 | 0.0187 |
|  | AD | 0.9441 | 1.9921 | 1.9537 | 0.0048 |  | 0.0002 | 0.0797 | 0.0061 |
|  | A | 1.4939 | 3.4659 | 2.3170 | 0.0047 |  | 0.0003 | 0.0333 | 0.0106 |
| P2 | ADR | 4.1095 | 4.8517 | 2.1249 | 0.0022 |  | 0.0022 | 0.0427 | 0.0126 |
|  | AD | 3.8892 | 5.3221 | 2.2011 | 0.0037 |  | 0.0004 | 0.0132 | 0.0077 |
|  | A | 4.2598 | 4.8350 | 2.0193 | 0.0053 |  | 0.0001 | 0.0067 | 0.0085 |
| CV (± %) |  | 28 | 24 | 29 | 41 |  | 93 | 72 | 38 |

Supplementary Table S1b. Enrichment with ^13^C in pea plant parts, supplied with the labelling solutions, and in triticale plant parts as intercrop at the end of a 90-day greenhouse pot experiment.

|  | Transfer | ^13^C atom%excess | | |  |  |  | | |
| --- | --- | --- | --- | --- | --- | --- | --- | --- | --- |
| Varieties | path | Pea | | |  |  | Triticale intercrop | | |
|  |  | Grain | Straw | Roots | Soil |  | Soil | Roots | Straw |
| Frisson | ADR | 0.0676 | 0.4623 | 0.4998 | 0.0011 |  | 0.0011 | 0.0132 | 0.0132 |
|  | AD | 0.0351 | 0.3508 | 0.3442 | 0.0013 |  | 0.0006 | 0.0089 | 0.0089 |
|  | A | 0.0629 | 0.7654 | 0.5794 | 0.0013 |  | 0.0006 | 0.0029 | 0.0029 |
| P2 | ADR | 0.1662 | 0.8060 | 0.8060 | 0.0010 |  | 0.0010 | 0.0051 | 0.0004 |
|  | AD | 0.1795 | 0.8881 | 0.8881 | 0.0011 |  | 0.0008 | 0.0008 | 0.0005 |
|  | A | 0.1820 | 0.8415 | 0.8415 | 0.0016 |  | 0.0006 | 0.0005 | 0.0008 |
| CV (± %) |  | 62 | 37 | 37 | 26 |  | 24 | 122 | 159 |

1. * Corresponding author: Tel.: + 49 5542 98 1598; e-mail: a.hupe@uni-kassel.de

   ** Current address: Landesbetrieb Landwirtschaft Hessen, Kassel, Germany [↑](#footnote-ref-1)
